# Functional characterization of 5′ untranslated region (UTR) secondary RNA structures in the replication of tick-borne encephalitis virus in mammalian cells

**DOI:** 10.1371/journal.pntd.0011098

**Published:** 2023-01-23

**Authors:** Laura Upstone, Robin Colley, Mark Harris, Niluka Goonawardane

**Affiliations:** 1 School of Molecular and Cellular Biology, Faculty of Biological Sciences and Astbury Centre for Structural Molecular Biology, University of Leeds, Leeds, United Kingdom; 2 School of Biological Sciences, University of Reading, Reading, United Kingdom; Oregon Health and Science University, UNITED STATES

## Abstract

Tick-borne Encephalitis Virus (TBEV) is an emerging flavivirus that causes neurological disorders including viral encephalitis of varying severity. Whilst secondary RNA structures within the 5′ untranslated regions (UTRs) of many flaviviruses determine both virus replication and pathogenic outcomes in humans, these elements have not been systematically investigated for TBEV. In this study, we investigated the role of predicted RNA secondary elements of the first 107 nucleotides (nts) of the viral genome forming the stem-loop A (SLA). Experiments were performed in replicons and infectious TBEV system. This region comprises three distinct structures: 5’ stem 0 (S0), stem-loop 1 (SL1) and stem-loop 2 (SL2). S0 was found to be essential for virus infection as mutations in the lower stem of this region significantly reduced virus replication. Point mutations in SL1 that preserved the Y-shape confirmation delayed viral RNA replication but did not abolish virus infectivity. Deletion of SL2 did not abolish infectivity but had a negligible effect on virus propagation. No correlation was observed between *in vitro* translation efficiency and virus infectivity, suggesting that the 5’UTR functions independently to virus translation. Together, these findings reveal distinct RNA elements within the 5′UTR that are essential for the stability and replication of viral RNA. We further identify changes in RNA folding that lead to altered TBEV infectivity and pathogenesis.

## Author summary

Tick-borne Encephalitis Virus (TBEV) is becoming endemic in regions previously unreported, including large areas of Europe and Asia. TBEV was also isolated in the UK in 2019, highlighting its potential as a concern to public health. TBEV is spread by ticks and causes severe and often fatal tick-borne encephalitis (TBE). No effective anti-TBEV therapeutics are available. TBEV circulates as three major subtypes that cause disease of varying severity. The reasons for the disparity in disease outcome are largely unknown. TBEV subtypes show high levels of nucleotide conservation at the 5′ non-coding (NC) region that folds to form a Y-shaped stem-loop A (SLA), which is conserved for all flaviviruses. Herein, we describe the identification of stem-loop regions in the SLA that are required for RNA replication and virus infectivity in cell culture, but do not influence viral translation. The observed virus attenuation suggested that the 5′ SLA likely serves as an essential regulatory element for binding to host factors that support viral replication and pathogenicity. These data highlight the complex interactions within specific TBE viral RNA conformations and reveal new targets for the development of much needed anti-TBEV therapeutics.

## Introduction

Tick-borne encephalitis virus (TBEV) is the causative agent of tick-borne encephalitis (TBE), a disease of the central nervous system (CNS) that leads to severe neurological complications, including fatal encephalitis [1,2]. TBEV is endemic in parts of Europe and Asia [2–4]) and is increasing its disease range due to expansion of its tick vector as a result of climate change [5–7]. Despite its continual emergence, no effective antiviral therapies are currently available for TBEV associated disease [3,8–10].

TBEV belongs to the genus *Flavivirus* in the family *Flaviviridae* [11], which can be further sub-divided into three closely related groups: European (TBEV-Eur), Far Eastern (TBEV-FE) and Siberian (TBEV-Sib) [12]. The recent increase in sampling and sequenced-based analysis now suggest further subdivision into genotypes 4 [TBEV-Bkl-1] and 5 [TBEV-Bkl-2])[13–16]. All TBEV strains show high similarity at the nucleotide (~82–85%) and amino acid level (92 to 96%) [16–18]. Despite this close homology, the clinical outcome of infection by different TBEV isolates ranges from asymptomatic (80–98% of cases) to encephalitis of varying severity [1,4]. The reasons for this variability remain largely unclear.

**Fig 1:**
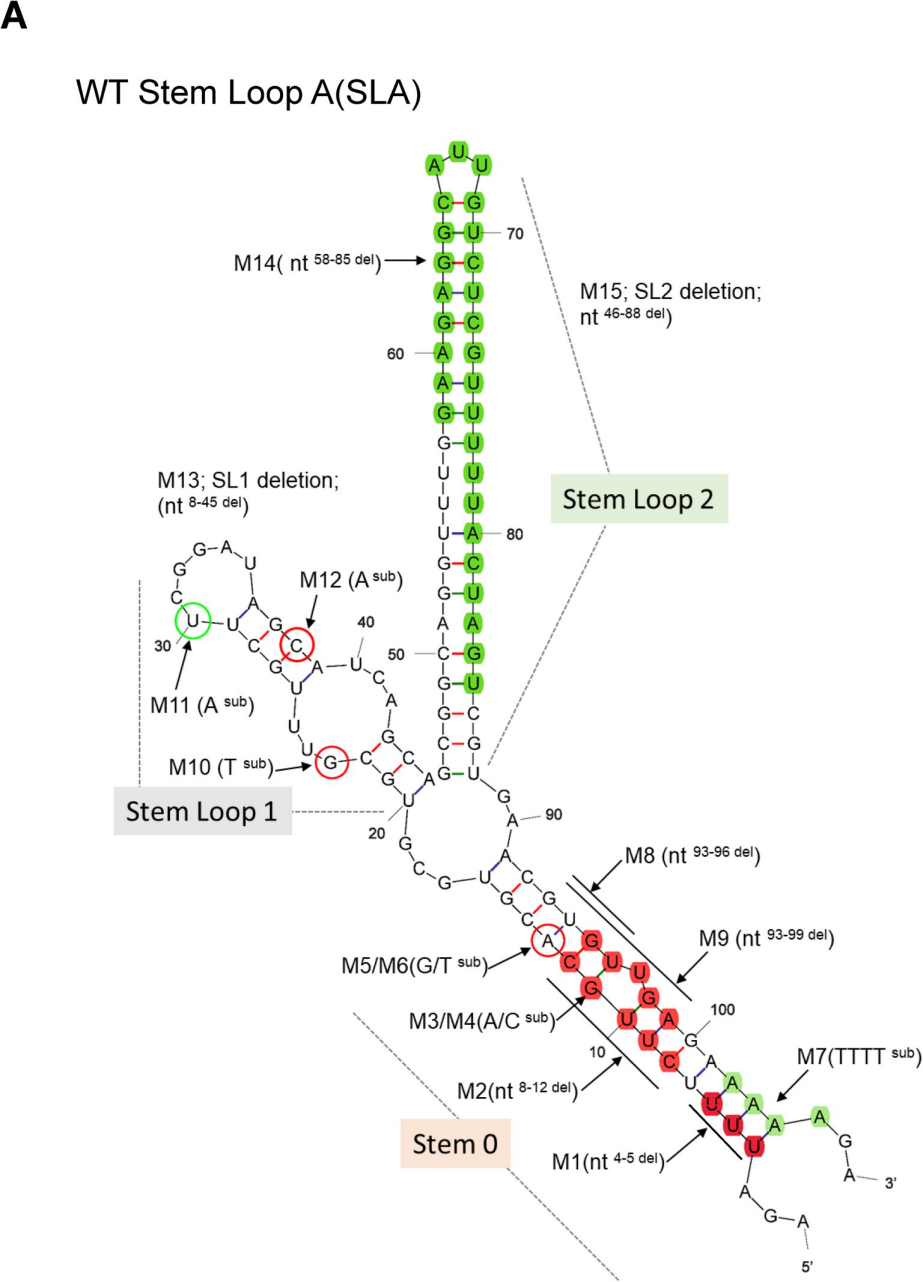
mFold structure of the 5′UTR of TBEV. (**A**) Computer-simulated predictions of WT TBEV with schematic representation of specific mutations within the SLA (arrows). Single nucleotide changes for M3-M6 and M12 (non-infectious) are circled in red. M11 (infectious) is in green. Double loop deletions (M16-M18) are not shown. Proposed sequence elements within Stem-Loop A (SLA) are labelled. Each deleted region is depicted by a solid line. Non-lethal mutants are highlighted in green. The first 107 nucleotides of the WT (GenBank accession: AF069066) region was assessed using Mfold (available on http://www.unafold.org/mfold/applications/rna-folding-form.php).

The TBEV genome is a positive-sense, single-stranded RNA (~11,000 nucleotides [nts]) that encodes a single polyprotein. This polyprotein is processed by viral- and cellular- proteases into three structural (C, M and E) and seven non-structural (through NS1-NS5) proteins. The latter comprise proteolytic and replicative enzymes, which form the viral RNA polymerase complex [11]. The TBEV genome is flanked by the 5′ and 3′ UTRs, which are crucial for the initiation of genome replication and the translation of viral proteins [19,20]. The 5’ and 3’ UTRs dimerize, leading to cyclization of the genome via the formation of a panhandle structure [21,22]. Secondary RNA structures within the 5’ and 3’UTRs regulate the rate of virus replication, RNA polarity, and genome encapsidation [19,23,24]. Y-shaped structures within three major thermodynamically stable structural elements of the 5’UTR have been predicted through simulations of secondary RNA folding for tick- and mosquito-borne flaviviruses (TBFV, MBFV) [25,26]. These include stem (S0) and two branching stem-loop structures 1 (SL1) and 2 (SL2) (Fig 1A). Although the role of individual elements within the 5′UTR have been investigated for MBFV, their role within the life cycle of other TBFVs remain largely undefined.

In this study, using TBEV as a model, we introduced deletions, point mutations and nucleotide substitutions into the 5′UTR of an infectious clone of TBEV and a replicon system. Herein, we report the essential roles of these RNA elements during viral RNA replication, with no accompanying roles in virus translation.

## Methods

### Cells

Porcine embryo kidney (PS) cells were grown in RPMI 1640 media (Gibco Life technologies) supplemented with 5% fetal bovine serum (FBS) at 37°C in a CO_2_ (5%) incubator. Unless otherwise stated, replicon-transfected PS cells were maintained in media supplemented with 2% FBS and 1% Penicillin-Streptomycin (Invitrogen).

### Construction of TBEV 5’UTR mutants

All plasmids contained the cDNA of TBEV-Sib strain Vs (GenBank accession: AF069066), under the control of a SP6 promoter, using intermediate plasmids or through the synthesis of *de novo* DNA fragments followed by Gibson assembly using NEBuilder HiFi (New England BioLabs [NEB]). Mutants more than 10 nucleotides (>10-nt) deletions (M13-18) were generated via string gene synthesis (GeneArt, Thermo Fisher Scientific) and assembled into the plasmid vector using NEB HiFi assembly.

DNA plasmids were amplified in *E*.*coli* DH5α (Promega) and purified with commercially available plasmid DNA isolation kits as per the manufacturer’s protocols (Qiagen). Point mutations and nucleotide substitutions were introduced into the infectious clone (IC) or TBEV replicon [17], using Q5 site-directed mutagenesis kits (NEB). All primers are shown in Table 1. Mutations were confirmed by sequencing.

**Table 1:** Mutants and primers used in the study.

Site of mutation	Mutation ID	Nucleotide position in the 5′UTR (5’→3’)	Predicted effect in the Y-shape structure	Primer sequence
**S0**	M1	4–6 (ΔTTT)	Shortened S0, preserved SL1/SL2	Fwd: TCTTGCACGTGCGTGCGT Rev: TCTGCGGTCTCTTTCGACAC
	M2	8–12 (ΔCTTGC)	Shortened S0, preserved SL1/SL2	Fwd: ACGTGCGTGCGTTTGCTT Rev: AAAATCTGCGGTCTCTTTCGAC
	M3	11 (G>A)	Preserved Y-shape SLA structure	Fwd: AGATTTTCTTaCACGTGCGTGC Rev: GCGGTCTCTTTCGACACT
	M4	11 (G>C)	Preserved Y-shape SLA structure	Fwd: AGATTTTCTTcCACGTGCGTG Rev: GCGGTCTCTTTCGACACT
	M5	13 (A>G)	Preserved Y-shape SLA structure	Fwd:ATTTTCTTGCgCGTGCGTGCG Rev: CTGCGGTCTCTTTCGACAC
	M6	13 (A>T)	Preserved Y-shape SLA structure	Fwd: ATTTTCTTGCtCGTGCGTGCG Rev: CTGCGGTCTCTTTCGACA
	M7	102–105 (AAAA>TTTT)	Preserved Y-shape SLA structure	Fwd: CGTGTTGAGAttttGACAGCTTAGGAG Rev: TTCACGACTAGTAAAAACG
	M8	93–96 (ΔGTGT)	Disrupted Y-shape structure	Fwd: TGAGAAAAAGACAGCTTAG Rev: GTTCACGACTAGTAAAAAC
	M9	93–99 (ΔGTGTTGA)	Disrupted Y-shape structure	Fwd: GAAAAAGACAGCTTAGGAG Rev: GTTCACGACTAGTAAAAAC
**SL1**	M10	23 (G>T)	Disrupted SL1 internal loop	Fwd: ACGTGCGTGCtTTTGCTTCGG Rev: GCAAGAAAATCTGCGGTCTC
	M11	30 (T>A)	Preserved Y-shape SLA structure	Fwd: TGCGTTTGCTaCGGATAGCAT Rev: CGCACGTGCAAGAAAATC
	M12	38 (C>A)	Shortened SL1	Fwd: CTTCGGATAGaATCAGCAGCGG Rev: CAAACGCACGCACGTGCA
	M13	Δ18–45 (SL1 Δ)	Deleted SL1, Long stem formation	GeneArt strings DNA fragment
**SL2**	M14	Δ58–85 (ΔPOWV like deletion in the SL2)	Shorten SL2	GeneArt strings DNA fragment
	M15	Δ46–88 (ΔSL2 deletion)	Long stem formation, disrupted Y-shape structure	GeneArt strings DNA fragment
**S0-SL2**	M16	Combined mutant: M1+M14	Shortened SL2, disrupted Y-shape structure	GeneArt strings DNA fragment
**SL1-SL2**	M17	Combined mutant: M13+M14	Long stem formation, disrupted Y-shape structure	GeneArt strings DNA fragment
**SL1-SL2**	M18	Δ20–29 and Δ37–92	Stem-loop formation, disrupted Y-shape structure	GeneArt strings DNA fragment

The construction of the TBEV replicons has been described elsewhere [17]. Briefly, most structural genes (nts 183–2386) were removed to make them non-infectious. The 5’UTR sequence and Stem-loops (SLs) 3 and 4 were retained in the C-terminus of the C-protein due to their requirement during the formation of secondary RNA structures essential for genome cyclization. Luciferase was cloned downstream of the 5’-end of the C-protein to provide a marker of both replication and translation as previously described [17]. Dinucleotides were modified (no CpGs and low UpA frequency[cu]) to enhance replication capacity [17,27]. Firefly luciferase contained no CpGs and low UpAs (termed luc-cu). To ensure correct polyprotein processing and NS1 translocation to the ER [28], a ribosome-stuttering 2A peptide from foot-and-mouth disease virus (FMDV) was incorporated downstream of the luc-cu sequence prior to the transmembrane (TM) region of E (22 codons).

Negative control firefly luciferase reporter replicons ΔAUG and NS5-GAA were constructed using Q5-site-directed mutagenesis according to manufacturer’s recommendations (NEB). Modification of the TBEV replicon construct has been described elsewhere [17]. Briefly, a ΔAUG replicon was generated by deletion of the start codon at position 133–135 (numbered according to the full-length sequence of strain Vs). Primers were as follows: ΔAUG-Fwd: GCCAGGAAGGCCATTCTG and ΔAUG-Rev: CCCCAGCTCTTGTTCTCC. NS5-GAA, which is deficient for RNA replication, was generated through mutation of the conserved GDD motif in the active site of NS5 to GAA (9652–9660), as previously described [29].

### Recovery of replicon RNA

DNA plasmids encoding relevant mutations were linearized at the *Sma*I restriction site downstream of the TBEV coding sequence and used as a template to produce full-length capped RNA using SP6 RNA polymerase (Promega). Each reaction (50 μL total volume) contained 1 μg of linearized DNA template and 40 units of SP6 RNA polymerase. Reactions were performed at 37°C for 3 h followed by treatment with RNase-free DNase I to remove template DNA. RNAs were purified using the RNeasy Mini Kit (Qiagen) to remove free nts, resuspended in RNase-free water (Invitrogen). RNA integrity was verified by agarose gel (1%) electrophoresis and quantified by spectrophotometry.

### RNA transfections

Transfections were performed using Lipofectamine 2000 (Invitrogen) as per the manufacturer’s recommendations. Briefly, 1 μg of SP6 transcribed RNA was complexed with transfection reagent in serum- and antibiotic-free culture medium for 10 min at room temperature (RT). Complexes were added to cell monolayers for up to 72 h. All transfections were performed in triplicate.

### Plaque assays

PS cells were transfected with SP6-transcribed WT or mutant TBEV RNA (1 μg RNA for 1.5 x10^5^ cells) using Lipofectamine 2000 (Invitrogen). Infectious supernatants were collected 24 to 72 hours post infection (hpi) and plaque assays were performed as previously described [4,17,30]. Briefly, aliquots of virus were diluted with serum-free RPMI 1640 and added to monolayers of PS cells for 1 h at 37°C. The inoculum was then aspirated, and plates were overlaid with RPMI 1640 supplemented with 2% FCS and 1% SeaPlaque Agarose (Cambrex, USA) for 5 days at 37°C for plaque formation. Monolayers were fixed with 4% formaldehyde and stained with 0.05% crystal violet. Plaques were counted and virus titers were expressed as log10 pfu/ml. All virus work was performed in a Biological Safety Level 3 (BSL3) laboratory. For TBEV growth kinetics, PS cells were infected with WT or mutant virus at an MOI of 0.1 for 1 h. Infected cells were then washed with PBS and incubated in complete medium (2% FCS) at 37°C. Supernatants from infected cells were collected at 0, 12, 24 and 48 hpi and frozen at -80°C prior to plaque assay. Experiments were performed in triplicate on two independent experiments.

### Luciferase assays

PS cells were seeded at 1.5 x10^5^ cells per well in 96-well plates (n = 3) and transfected with *in vitro* transcribed TBEV replicon RNA (100 ng/well) and 10 ng of *Xba*I linearized pTK-Ren (Promega) using Lipofectamine-2000 (Thermo Fisher Scientific). Cells were harvested in passive lysis buffer (Promega) at 12 to 72 hpt. Luciferase activity was measured using the Dual Luciferase reagent kit and GloMax multi detection system (Promega). pTK-Ren (Promega) expressing Renilla luciferase (RLuc), was included as an expression control. Quantitative data were obtained from three independent experiments.

### Immunofluorescence assay (IFA)

Transfected cells cultured in 96-well plates (1.5 × 10^5^ cells/well, n = 3, Sigma), were fixed in 4% paraformaldehyde (Applichem GmbH) and permeabilised in PBS-T (0.1% v/v Triton X-100 in PBS) for 5 min. Cells were blocked in PBS-T containing 5% w/v BSA for 10 min and probed with rabbit-anti-NS5 antibodies [17] in PBS-T, 1% w/v BSA for 1 h at room temperature. After washing with PBS, cells were stained with 1:1000 fluorochrome-conjugated Alexa Fluor 488 (Life technologies, A32790) secondary antibodies in PBS-T, 1% w/v BSA for 1 h at room temperature in the dark. Microplates were analysed using an EVOS microscope (Scale bar: 100 μm). Images were obtained from two independent experiments.

### *In vitro* translation

TBEV replicons were derived from the cDNA clone of TBEV Siberian subtype Vs, the construction of which has been described elsewhere [17,20,30]. Firefly luciferase was cloned downstream of the 5′UTR and the sequence encoding the C-protein (codon 41) was modified to contain no CpGs and a low UpA frequency (luc-cu) to enhance replication capacity [4,27]. The self-cleaving 2A peptide from Foot-and-Mouth Disease virus (FMDV) was incorporated downstream of the luc-cu sequence prior to the transmembrane (TM) region of E (22 codons), to ensure correct translocation of the downstream NS1 protein into the ER. NS5-GDD was mutated to GDD in the RNA-dependent-RNA-polymerase (RdRp; NS5) [17,29], as a translation-competent, replication- deficient control.

TBEV transcripts were analysed for translation efficiency in cell-free systems, which contained only the minimal components necessary for translation. Rabbit reticulocyte lysate (Promega) was designed with 2 μg of transcript RNA. The cell-free system was as follows: 70 μl cell-free lysate, 2 μl AA-Met, 2 μl AA-Leu, 2 μl RNAsin, 2 μg RNA transcript, and water to a final volume of 100 μl. Reactions were gently vortexed and incubated at 30°C for 30, 60 and 90 min prior to the measurement of luciferase activity. Samples were assayed in triplicate and quantitative data were obtained for three independent experiments.

### Statistical analysis

Statistical significance was determined using GraphPad Prism. Data were compared using a one-way ANOVA with Bonferroni’s correction.

## Results

### Secondary RNA structure mutants

Linear and cyclization models of the secondary RNA structures of flavivirus 5′-3′ UTRs have been previously described [22,31]. The linear model can predict the independent folding of the UTRs, whilst the cyclization model proposes interactions between the 5′UTR and 3′UTR, resulting in genome cyclization and the formation of a long dsRNA stem. The latter has been confirmed experimentally [21,32]. Using the linear model, a conserved Y-shaped conformation was predicted for all 5′UTRs [12,26]. This contained a double-stranded stem (stem zero, S0) split into two stem-loop (SL) structures, SL1 and SL2 (Fig 1A) This folds independently of other downstream RNA structures and the dsRNA cyclization stem in the cyclization model [19,32].

**Fig 2:**
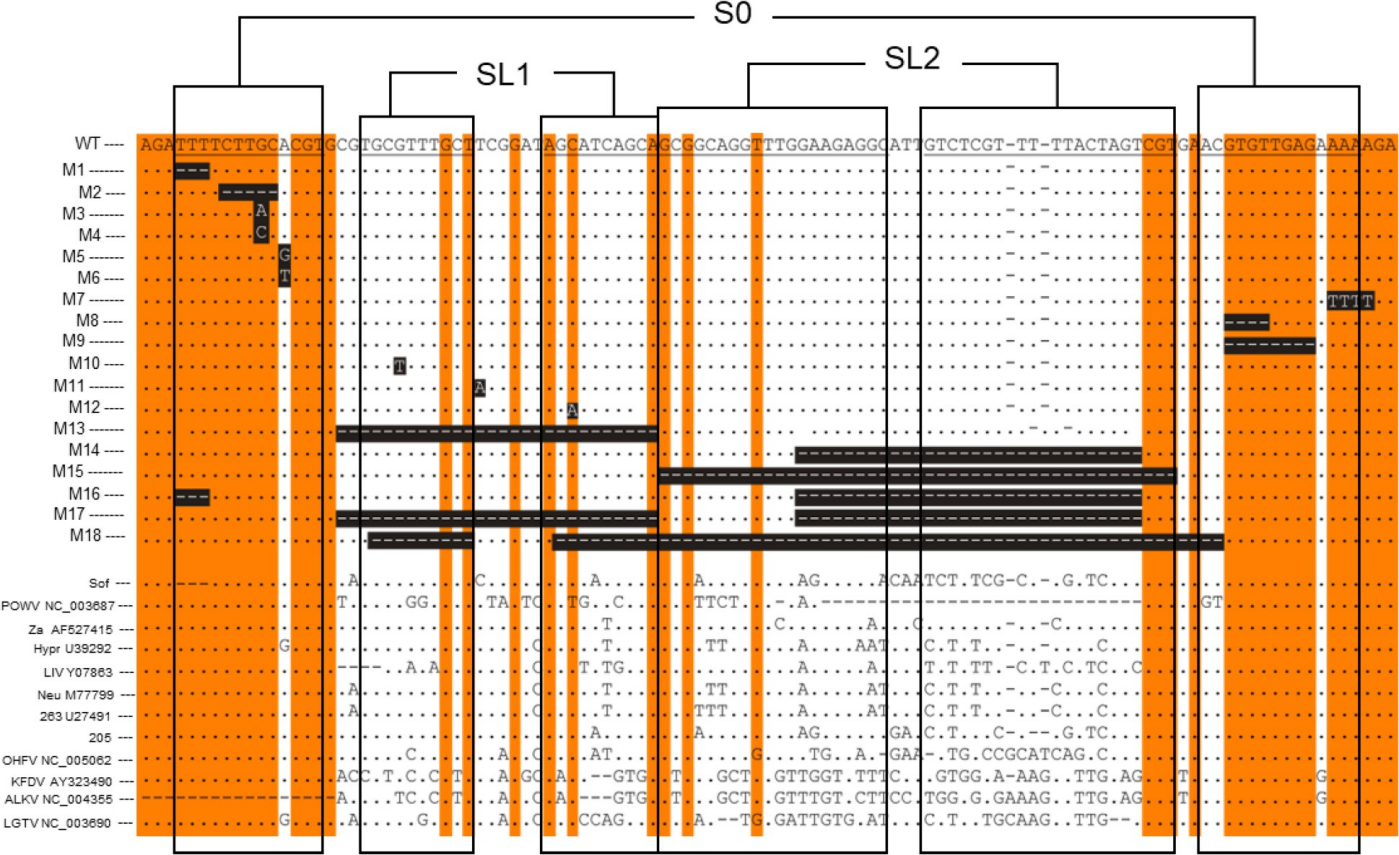
Alignment between the 5′UTRs of tick-borne flaviviruses and constructed mutants (M). Accession numbers are indicated. Reference sequences for Sof [44] are shown. The top line depicts Vs virus that was used to produce the infectious clone. Conserved regions are highlighted in orange. Elements of the RNA secondary structures are specified as S0, SL1, and SL2 within SLA. Deletions and substitutions are highlighted in white letters within black boxes. Numbers in front of the 5’UTR mutants correspond to the numbers in Fig 1.

We performed mutagenesis to reveal the role of the Y-shaped structural element during virus replication. mFold-[33] simulated structures for 5′UTR mutants (WT: Accession: L40361 and M1-M18) are shown in S1 Fig, containing mutated 5’UTRs aligned with corresponding sequences of other TBFV isolates. Boundaries of the stable secondary RNA structures predicted for the mutated 5′UTR (Fig 1) were superimposed on this alignment (Fig 2).

Deletions in M1 were designed to simulate naturally occurring deletions in the Sofjin (Sof) strain (S1B Fig, M1). These were predicted to shorten S0 but preserve SL1 and SL2. M2 mutant was designed to assess the roles of 5 highly conserved nucleotides in S0 that form the bulge of the stem. Deletions in this region led to a shorter S0 in mFold predictions. M3-M6 contains point mutations (nt) at positions 11 and 13, and nt-11, a region highly conserved amongst isolates (Fig 2). In M7, AAAA between nucleotides 102–105 of the 5’UTR at the base of S0 were replaced by TTTT (S1B Fig, M5). M8 and M9 contain deletions were in highly conserved regions of S0 (Figs 2 and S1B), predicted to ablate the original Y-shaped structure, whilst rearranging conformations into 3 short SLs and one long stem region.

Mutations M10-M12 were designed to test the SL1 where M10 and M11 (S1A Fig, M11) are located in the internal and terminal loops, respectively. M10 and M11 preserve the Y-shaped structure, whilst highly conserved nt changes in M12 shorten the SL2. SL1 is deleted in M13 resulting in a single long stem as opposed to a Y-shaped structure (S1C Fig).

Mutant M14 was designed to simulate a naturally occurring deletion in Powassan virus (POWV). This shortens SL2 whilst preserving both S0 and SL1 (S1C Fig, M14, and 2). M15 removes SL2 in its entirety, leading to an extended stem with preserved S0 and SL1.

M16, M17 and M18 contain deletions in two or more elements within SLA. M16 was designed by combining M1 and M14, whilst M17 contains M13 and M14. M18 contained a deleted SL2 and the stem region of SL1. All three mutants manifest as a disrupted Y-structure with a shortened SL1 and SL2 (S1D Fig).

### RNA replication is regulated by elements preserved at the 5′UTR of the TBEV genome

**Fig 3:**
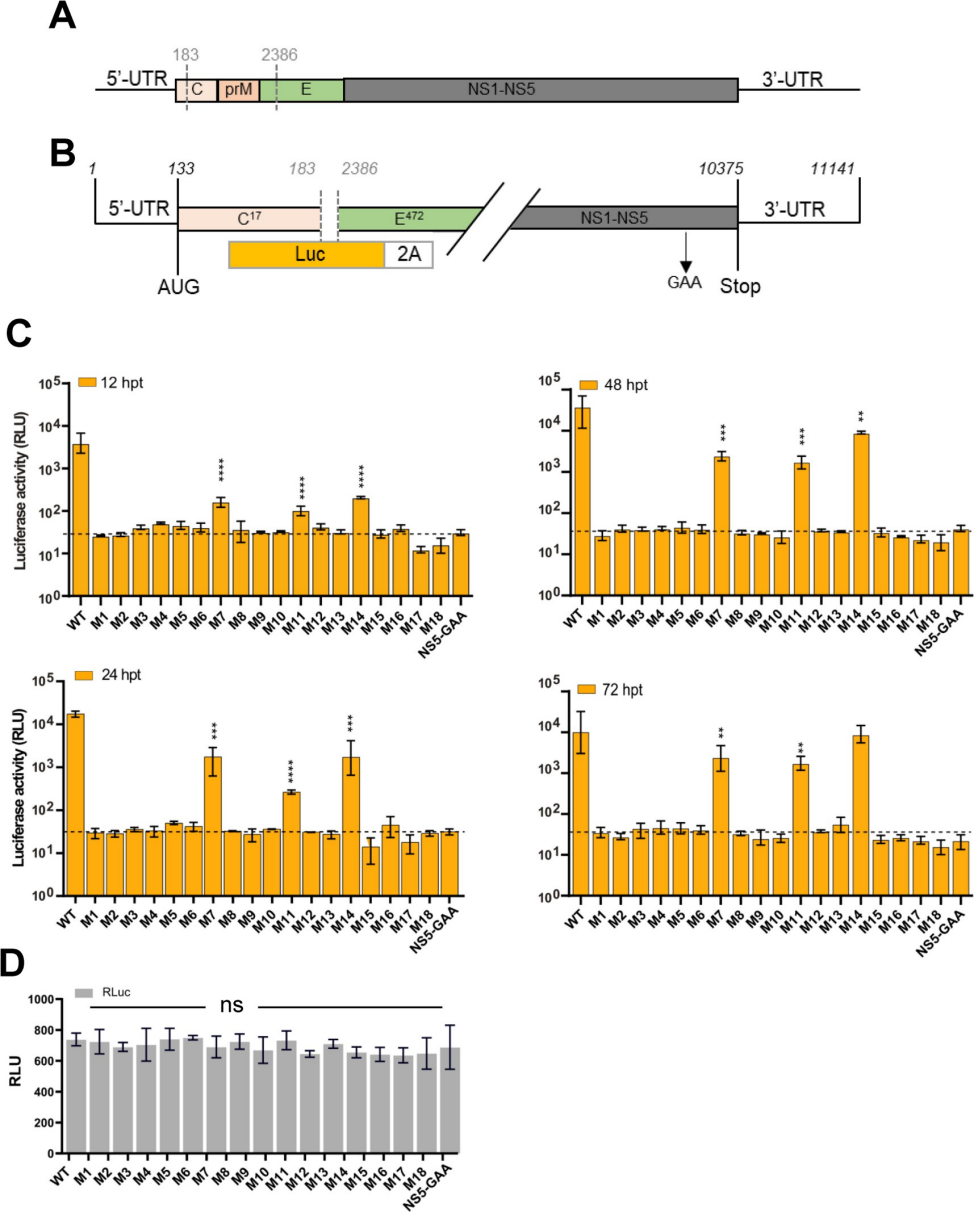
Replication kinetics of the 5′UTR mutants. (**A**) Schematic representation of the TBEV genome (**B**) and TBEV replicon in which the structural genes were replaced with firefly (cu) luciferase, and Foot-and-mouth disease virus 2A site (FMDV-2A). The NS5 polymerase inactive site GAA is indicated. (**C**) Luciferase assays in PS cells co-transfected with WT or 5′UTR mutants over the indicated time course (12–72 h post-transfection [hpt]). (**D**) Representative quantification of pTK-Ren (Renilla) performed 24 h post-transfection (hpt), showing nonsignificant (ns) differences between mutants. Bar heights are the mean ± SEM of three biological replicates. Assays were performed in triplicate. *P = <0.01, **P = <0.001, ****P = <0.0001 versus WT determined using a one-way Anova with Bonferroni’s correction.

To more precisely map the structural elements within the SLA that are responsible for genome amplification, deletions and mutations were introduced into the first 107 nt, forming elements S0, SL1 and SL2. We initially tested their ability to replicate using TBEV luciferase replicons in PS cells (Fig 3C). Cells were harvested at defined time points post-transfection (12–72 h), and firefly luciferase activity translated from mutant replicon RNA, was measured over a 72 h period. Deletions or point mutations within S0 were not tolerated (Fig 3C), and completely abolished RNA synthesis (M1-M6 and M8-M9). In contrast, M7 bearing a 4-nt TTTT substitution at position 102–105 that simulates the single-stranded trinucleotide AGA (position 1–3 of the 5′UTR) in S0 was replication-competent, albeit at a substantially reduced rate compared with WT: At 12 hpt, M7 showed a ~20-fold reduction in replication. Replication increased at 24 h and was maintained to competent levels thereafter (48–72 h).

A point mutation in SL1 (M11) resulted in a 2-log reduction in replication compared with WT during the first 24 hpt. However, M11 replication was elevated over the course of 48 to 72 hpt (Fig 3C). No correlation was observed between substituted nucleobases and the rate of replication for all assayed mutants. Large deletions in the SL2 did not abolish replication (Fig 3C, M14), but relatively lower rates of replication were observed at 12 to 24 h, reaching similar levels to WT at 72 hpt. Rluc levels (internal control) were comparable for all transfected mutants, with representative quantification at 24 hpt shown on Fig 3D. This confirmed that the differences observed directly reflected nucleotide changes.

### SLA is an essential cis-acting element for TBEV

TBEV replicons do not produce infectious virus progeny, meaning the full effects of 5′UTR mutations on the virus life cycle cannot be evaluated. To replicate other life cycle stages, we used a full-length cDNA clone of TBEV [30] and generated recombinant infectious RNAs [17,20], with desired replication-competent 5’ SLA mutations. Equal quantities of *in vitro* transcribed RNAs corresponding to WT and M7, M11, and M14 mutants were transfected into PS cells and infectivity was assessed by immunofluorescence (IF) using specific anti-NS5 antibodies. The recovery of infectious viruses were quantified by plaque assays of cell supernatants at 24–48 hpi.

**Fig 4:**
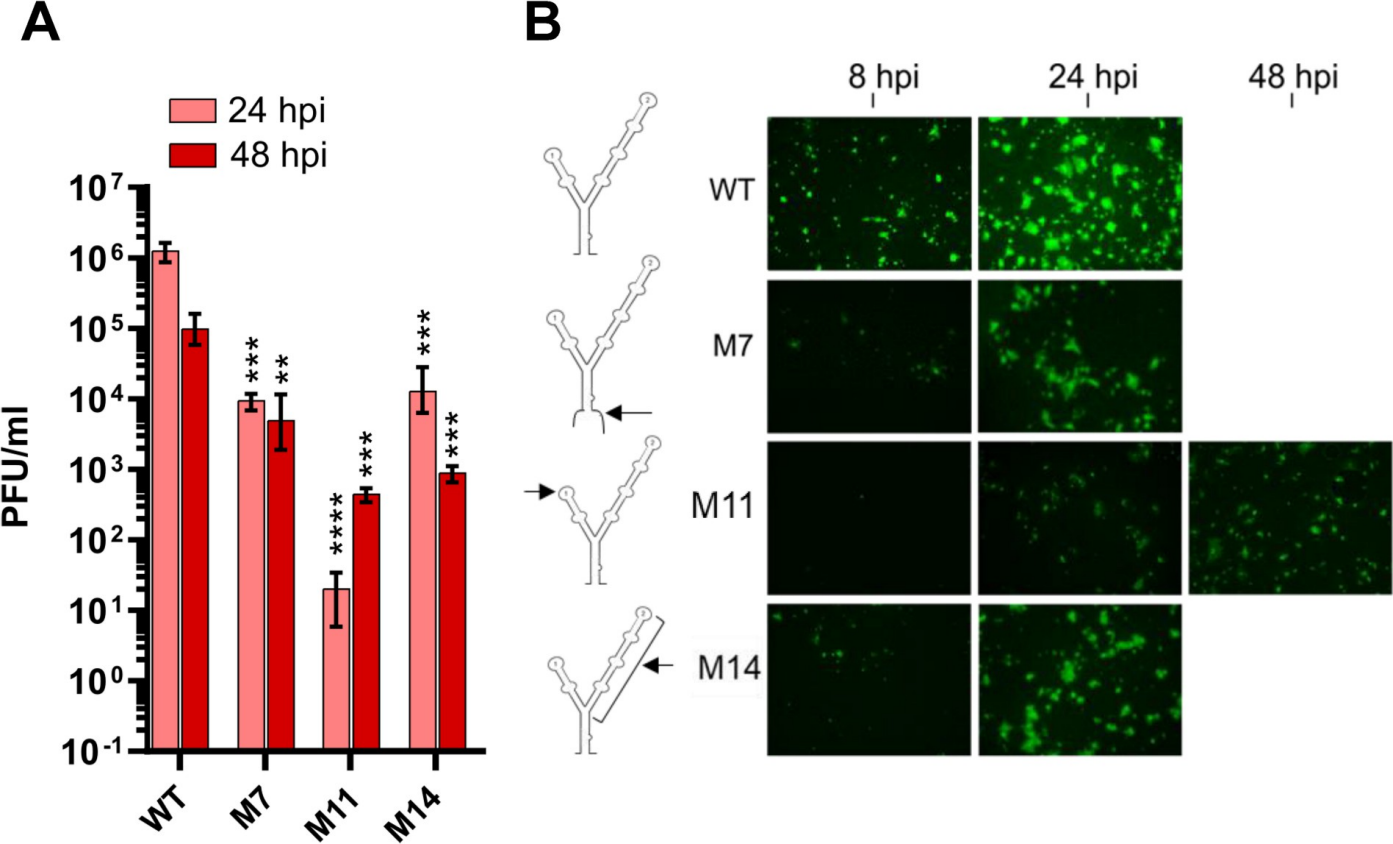
Infectivity of mammalian cells by 5′UTR mutants. (**A**) Quantification of plaque assays from PS cell supernatants infected with 5’UTR WT and mutant viruses 24 h post-infection (hpi). **P<0.001, ***P<0.001, ****P<0.0001 versus WT from 24 or 48 hpi determined in two biological replicates (n = 3 technical repeats). Data were analysed using a one-way Anova with Bonferroni’s correction. (**B**) Expression of NS5 in PS cells transfected with WT or mutant viruses assayed over the indicated timeframe. Schematic representation of the mutants within the Y-structure are shown (arrows).

PS cells transfected with WT RNA transcripts were virus positive after 12 h and nearly ~100% of the monolayer was antigen-positive at day 1 (Fig 4A). Further characterization of the mutants by IF revealed that the SL1 mutant (M11) was antigen-negative at 12 h, but showed virus-specific fluorescence over the next 48 h, albeit at lower levels than WT.

A total of three mutants were infectious by plaque assay: one each in SL1 (M11), SL2 (M14) and at the base of SLA (M7). When recovered, infectious mutants were compared with WT virus (Fig 3B). Substitutions in SL1 showed substantial attenuation in infectious titer (at least 4 orders of magnitude) at 24 hpi. However, a remarkable recovery was observed at 48 hpi, with infectious titers reaching ≥10^3^ PFU/ml. M7 and M14 showed a 2-log and log reduction in infectious titer after 24 and 48 hpi, respectively, compared to WT. M14 was infectious and isolated viruses replicated to levels comparable to WT. Collectively, these data suggest that mutation of the Y-structure influences virus replication and base-pairing at SLA, with regions within SL1 required for viral genome replication.

### Effects of SLA on virus protein synthesis

**Fig 5:**
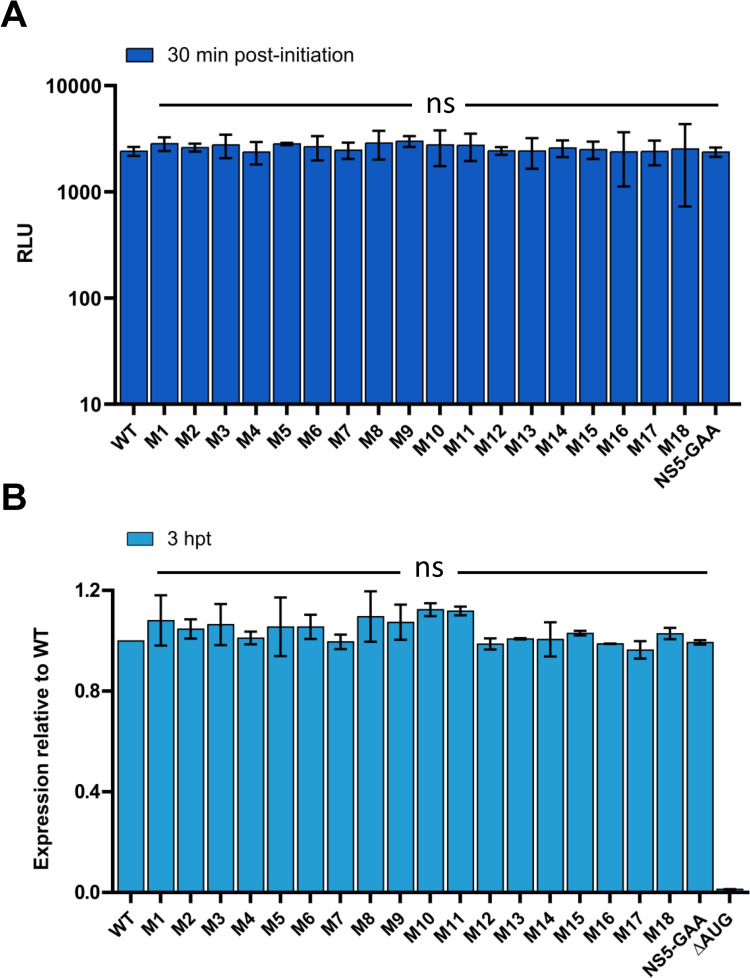
Translation of WT and SLA-mutant replicons. (**A**) Translation rates of 5′UTR mutants in cell-free translation assays 30 min post initiation. NS5-GAA RNA was evaluated as a negative control. (**B**) Translation of input mutant RNA in PS cells at 3 hpt. Normalized luciferase levels are shown relative to WT. Replication-competent NS5-GAA RNA and ΔAUG, evaluated as a negative control. Data were derived from three biological replicates; error bars show standard deviation. ns indicates no significant difference from WT. Data were analysed using a one-way Anova with Bonferroni’s correction.

As some mutants were unable to initiate infection in cell culture (Fig 4), we next examined their effects on the initiation of translation. The TBEV genome is translated in a cap-dependent manner, in which the small 40S subunit of the ribosome scans SLA and SLB, reaching the initiator AUG codon [21,29]. To investigate whether components of the SLA play a role in translational regulation, we assayed translation in cell-free assays using rabbit reticulocyte lysates to determine the capacity of WT and *SmaI* linearized replicon SLA-mutant RNAs to direct polyprotein synthesis. Translation efficiencies were measured through luciferase activity relative to WT (Fig 5A). Replicons with a mutated SLA showed equivalent levels of translation to WT in cell-free reticulocyte lysates, indicating that modifications to the 5′UTR had no effect on translation initiation.

For further clarification, we investigated replication-deficient RNA transcripts in cell culture. To achieve this, equal amounts of mutant RNA were transfected [22,29,32] and translation levels of input RNA were monitored for 3 h (Fig 5B). All 5’-SLA mutants exhibited equivalent levels of translation to the WT construct, which contrasted the luciferase measurements observed beyond 24 hpt (Fig 2C). Collectively, these data confirm that deletions or mutations in the TBEV 5′UTR do not influence the translation of input RNA.

## Discussion

RNA structures play an essential role in the host adaptability of flaviviruses. All flavivirus 5′ NC sequences form secondary RNA structures that are critical for completion of the virus life cycle. Notable differences in the length of the side loop and size of the bulge exist for the Y-structure in the SLA of the 5’UTR between tick- and mosquito-borne viruses [25,34]. These variations likely dictate individual virus pathogenicity. The 5′ UTR of the dengue virus (DENV) is the most well-studied of all known human pathogenic flaviviruses. The topology of the DENV 5′ UTR is essential for efficient cyclization and viral replication [22,23,35,36]. The 5′ UTR of DENV contains a Y shaped stem loop A (SLA) structure, which resembles that of MBFV and TBFV. Elements within the SLA (namely SL1-2 and S0, Fig 1) have been characterized for DENV, West Nile virus and ZIKA [1,10,35–37]. Disruption in the 5’ RNA elements (SLA and SLB) influence virus pathogenicity. The 5’ NC sequences, SLB, SL3 and SL4 and the 3’UTR and 5′UTR sequences have been characterized in TBEV reporter systems [21,32] but not in infectious virus [38]. Systemic characterisation of RNA secondary structures of the 5′ UTR are critical to understand the function and regulation of TBEV pathogenicity.

We investigated the biological significance of SL1, SL2 and S0 in the SLA of tick-borne flavivirus 5′RNA, using TBEV as a model. A total of 18 distinct nucleotide changes were systematically introduced into regions of the SLA (S1 Fig). Mutants were individually predicted to change, disrupt or preserve the overall computer-simulated stem and loop structures of the SLA respectively, likely through affecting local base-pairing within elements.

Three of the mutants were replication-competent in the context of the TBEV replicon (Fig 3C), which is unable to generate infectious viruses. One of these mutants (M11) contained a point mutation in SL1 and showed significant reduced RNA replication. Similar to POWV, TBEV SL2 was able to tolerate deletion of two thirds of this region (M14), with a significant reduction in RNA replication. Unlike the conserved regions that serves as important functional elements, naturally occurring mutations would not be expected to substantially affect virus replication. Neither point mutations nor deletions in the S0 showed viable replication (e.g., M1-M6). M7, which leads to alterations in the trinucleotide AGA (position 1–3 of the 5’UTR) during the formation of S0, did not abolish replication, but led to a >log reduction in RNA amplification. All three replication-competent mutants had a preserved S0 and SL1 (Fig 1B), suggesting they are indispensable for RNA replication.

We next assessed the effects of mutations within S0, SL1, and SL2 on full-length TBEV IC. Virus infectivity was assessed in PS cells by plaque assay (Fig 3A). SL2 deletion mutants, M14 and M7 (at the base of S0), resulted in a significant attenuation of infectivity, which like WT virus, peaked at 24 hpi. In contrast, M11 showed delayed infectivity at 24 hpi. Further analysis using NS5 expression as a surrogate for virus infection (Fig 3A), revealed a delay in virus propagation in M11 compared with WT and M7/M14, indicating that SL1 is essential for the initiation of virus infectivity in vertebrate cells whilst SL2 is not. Mutations in S0 were lethal for TBEV. Indeed, S0 and SL1 were previously shown to be essential for RNA replication and infectivity for other flaviviruses [22,25]. For SL1, we speculate that mutations within the stem region (M12) destabilize loops that interact with important regulatory proteins. Conservation of the Y-structure SLA conformation in positive-sense RNA viruses highlights their importance during the virus life cycle, including SARS-CoV-2 [39–42].

To explain the loss or delay in infectivity associated with recovered 5′UTR mutants, we examined their effects on virus translation through *in vitro* cell-free assays and in TBEV replicon systems using a ΔAUG construct that is competent in translation but unable to replicate. The latter has been used to characterize translation in the TBEV 5’-3’ NC region [29,32]. Mutants that changed the Y-structure, including non-replicative and non-viable mutants, remained translationally active (Fig 5). A correlation between virus viability and translation within the SLA sequence was therefore, not observed. Furthermore, highly conserved elements within the 5′UTR (such as S0) appeared to serve as crucial functional elements (cis acting) for the initiation of virus replication as mutations in this region directly influenced RNA replication. This was exemplified by M7, which altered the base-pairing within S0 (Fig 1B). Alterations in SL1 led to restricted virus growth in mammalian cell culture (Fig 3). Since this element forms projectile RNA loops in the Y-structure, SL1 is likely to enhance RNA binding to host cell factors and/or other regions of RNA required for virus replication. Moreover, large stem regions that were non-essential may be required for the stability of SLA (M14; SL2 deletion attenuated virus growth). This is in agreement with previous observations showing that POWV strains of the tick-borne flavivirus group lack the corresponding 5’ NC sequence of this structure [43]. However, the evolutionary conservation of SL2 provides clues to its role within invertebrate hosts (ticks). The region proceeding the dsRNA S0 within SLA shows higher conservation amongst all flaviviruses forming, SL3 [21,22,26] (or SLB) in the linear model, and the dsRNA stem in the cyclization model. This is predicted to mediate the interaction between the 5’UTR and 3’UTR.

In summary, we have identified elements that directly (S0) or indirectly (SL1, SL2) regulate the initiation of TBEV replication. Importantly, virus attenuation was predominant following alterations in the extended stem-loop regions (SL1 and SL2) in mammalian cell culture, which offers the opportunity for exploitation for live attenuated vaccine strains. Crucially, identifying sequences within SLA (e.g., nt 58–85) that lead to host restriction, suggests that differential factors may support viral replication and associated cell tropism. The sequences described can be utilized to identify key host factors governing viral replication and novel targets for much needed antivirals against flaviviruses.

## Supporting information

S1 Fig Secondary RNA predictions for the mutated 5’UTR. For each mutant, regions of 107 nucleotides were assessed using Mfold (available on http://www.unafold.org/mfold/applications/rna-folding-form.php). **(A)** Mutants that resulted in infectious virus production (M7, M11 and M14). The region(s) of the mutation is highlighted in green. (B) Stem 0 (S0) mutants, (C) SL1 mutants and (D) SL2 (M15) and combined mutants (Table 1). Point mutations are shown in arrows.
(PDF)
