# Supplementary figures and images for: Functional characterization of 5′ untranslated region (UTR) secondary RNA structures in the replication of tick-borne encephalitis virus in mammalian cells
Source: PLoS Negl Trop Dis. 2023 Jan 23;17(1):e0011098. doi: 10.1371/journal.pntd.0011098 (PMC9894543; doi:10.1371/journal.pntd.0011098)

A

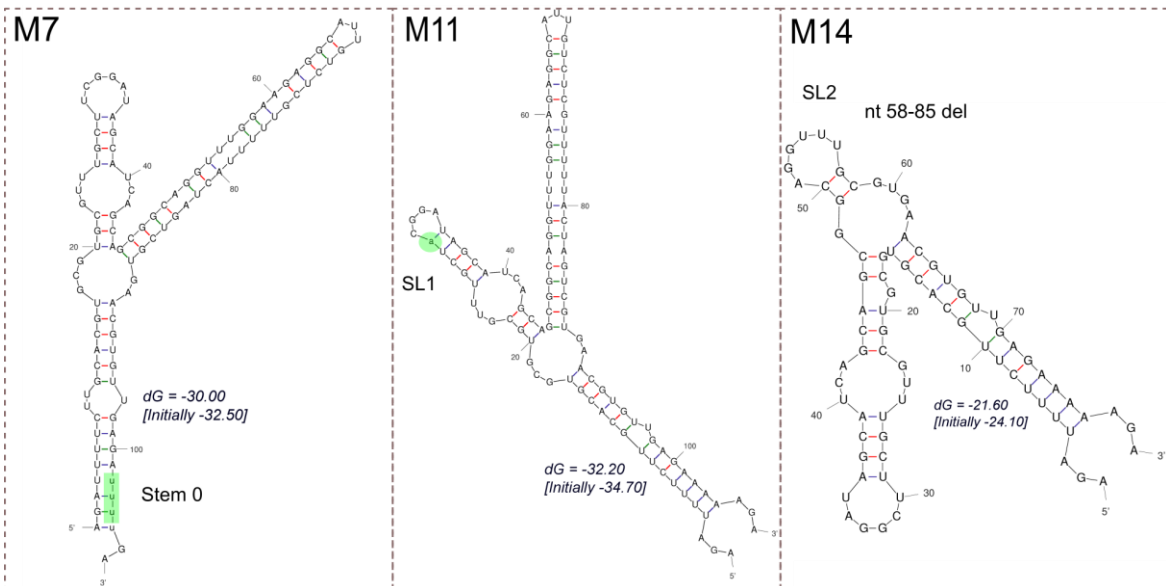

B

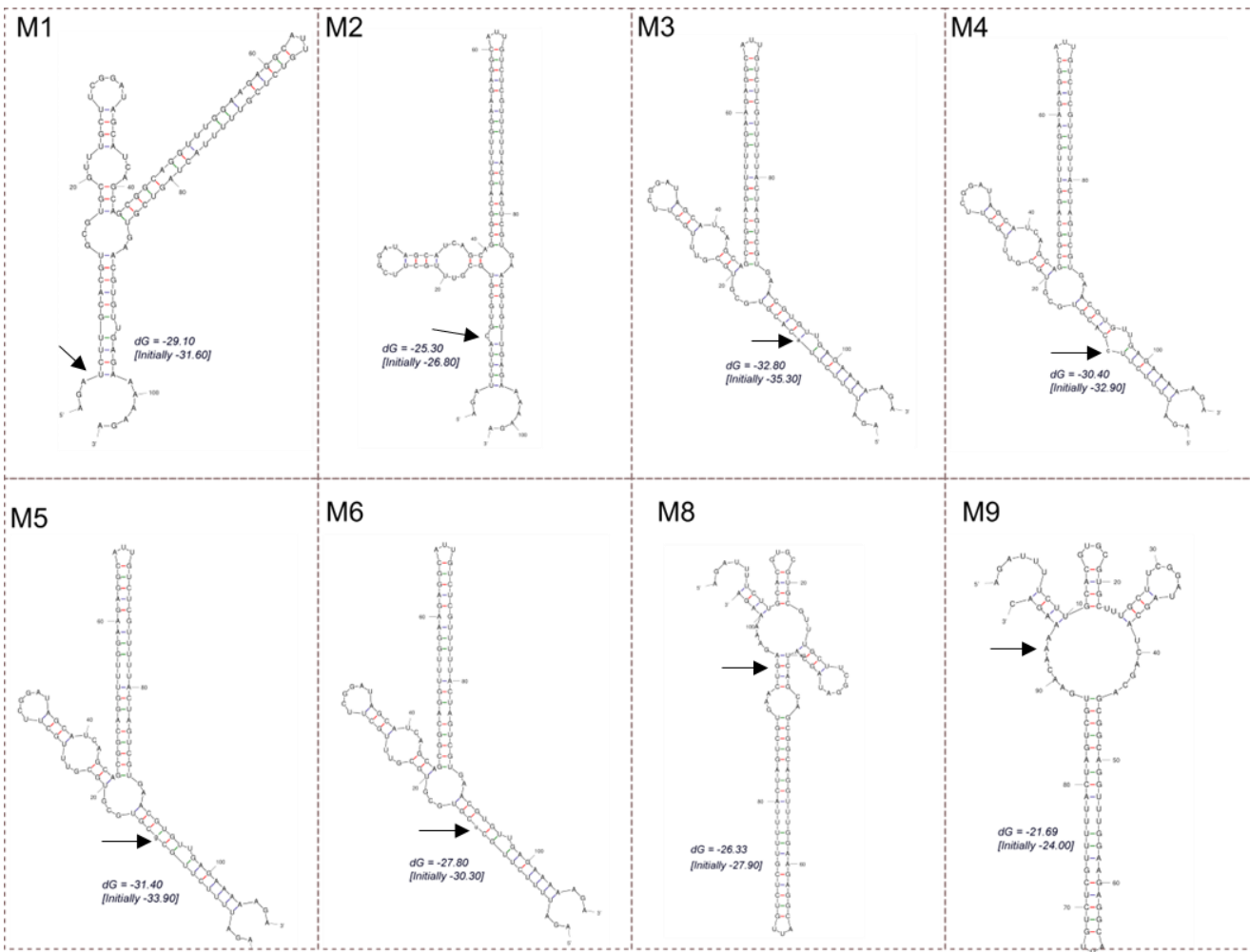

C

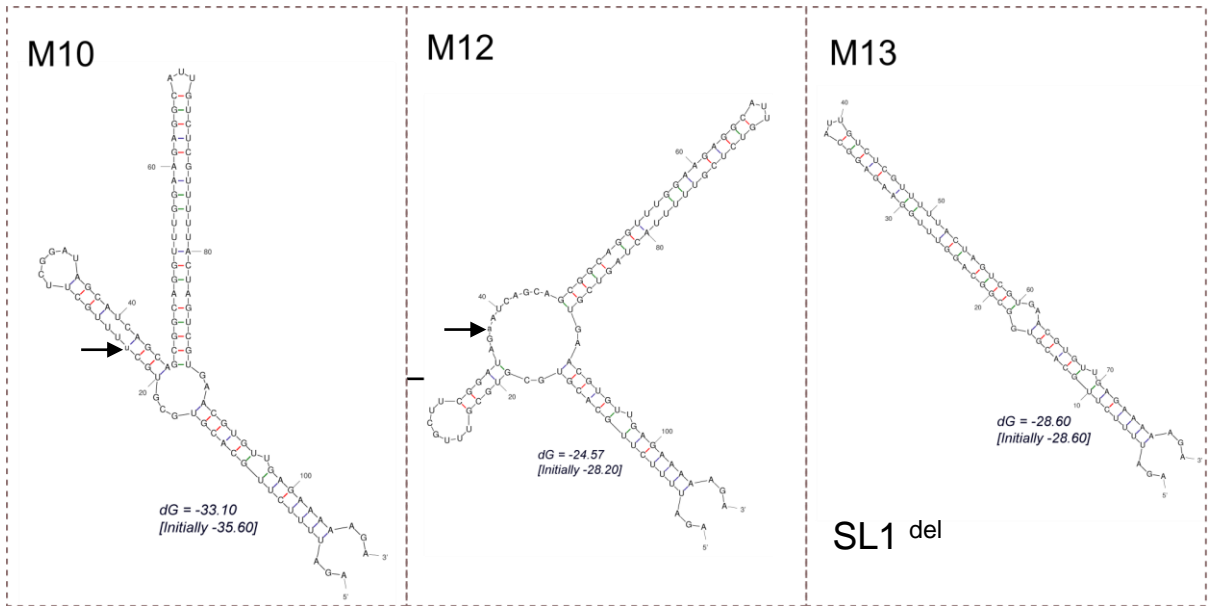

D

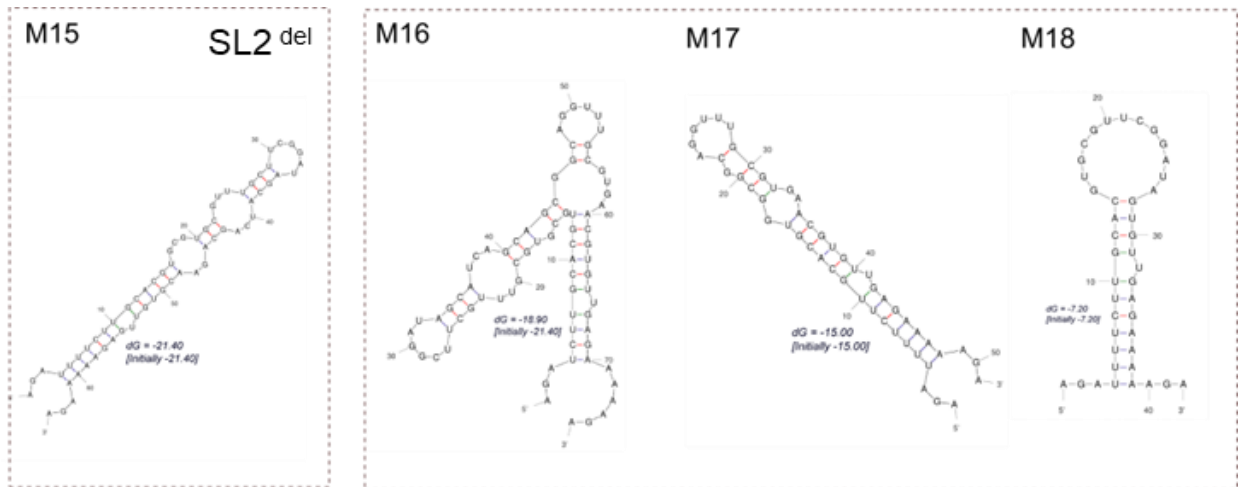

Supplement: S1 Fig — For each mutant, regions of 107 nucleotides were assessed using Mfold (available on http://www.unafold.org/mfold/applications/rna-folding-form.php). (A) Mutants that resulted in infectious virus production (M7, M11 and M14). The region(s) of the mutation is highlighted in green. (B) Stem 0 (S0) mutants, (C) SL1 mutants and (D) SL2 (M15) and combined mutants (Table 1). Point mutations are shown in arrows. (PDF) [file pntd.0011098.s001.pdf]
